# Test-retest, inter- and intra-rater reliability of the flexicurve for evaluation of the spine in children

**DOI:** 10.1590/bjpt-rbf.2014.0139

**Published:** 2016-01-19

**Authors:** Juliana A. Sedrez, Cláudia T. Candotti, Maria I. Z. Rosa, Fernanda S. Medeiros, Mariana T. Marques, Jefferson F. Loss

**Affiliations:** 1 Programa de Pós-graduação em Ciência do Movimento Humano, Escola de Educação Física, Universidade Federal do Rio Grande do Sul (UFRGS), Porto Alegre, RS, Brazil; 2 Departamento de Fisioterapia, Escola de Educação Física, UFRGS, Porto Alegre, RS, Brazil

**Keywords:** child, spinal curvatures, reliability studies, physical therapy, rehabilitation

## Abstract

**Introduction:**

The early evaluation of the spine in children is desirable because it is at this stage of development that the greatest changes in the body structures occur.

**Objective:**

To determine the test-retest, intra- and inter-rater reliability of the Flexicurve instrument for the evaluation of spinal curvatures in children.

**Method:**

Forty children ranging from 5 to 15 years of age were evaluated by two independent evaluators using the Flexicurve to model the spine. The agreement was evaluated using Intraclass Correlation Coefficients (ICC), Standard Error of the Measurement (SEM), and Minimal Detectable Change (MDC).

**Results:**

In relation to thoracic kyphosis, the Flexicurve was shown to have excellent correlation in terms of test-retest reliability (ICC_2,2_=0.87) and moderate correlation in terms of intra-(ICC_2,2_=0.68) and inter-rater reliability (ICC_2,2_=0.72). In relation to lumbar lordosis, it was shown to have moderate correlation in terms of test-retest reliability (ICC_2,2_=0.66) and intra- (ICC_2,2_=0.50) and inter-rater reliability (ICC=0.56).

**Conclusion:**

This evaluation of the reliability of the Flexicurve allows its use in school screening. However, to monitor spinal curvatures in the sagittal plane in children, complementary clinical measures are necessary. Further studies are required to investigate the concurrent validity of the instrument in order to identify its diagnostic capacity.

## BULLET POINTS

- The Flexicurve has test-retest, intra- and inter-rater reliability confirmed.
- The Flexicurve can be used for evaluating spinal curvatures in children.
- The Flexicurve can be used in school screening to detect postural alterations.

## Introduction

Spine evaluation is essential both for monitoring^1^ and diagnosing vertebral alterations, with radiography being the most appropriate method for both processes^2^. However, radiography entails exposure to undesirable radiation levels, so non-invasive methods are of great benefit^3^ because of their lower cost, fewer technical difficulties, and the absence of exposure to ionizing radiation^4^.

Among the non-invasive methods is the Flexicurve, a flexible ruler that was first described by Takahashi and Atsumi^5^. The Flexicurve allows measurements in the sagittal plane and can be used in several surroundings^6^. The psychometric properties of this instrument have been described for use with adults^7^, and it is seen as a low-cost and quick evaluation instrument^8^.

School-age children have a significant prevalence of postural imbalances^9^, and the early detection of postural changes can be important. The Flexicurve instrument can be a screening tool because it is easily accessible for the school environment. This is even more important when considering that schoolchildren are likely to exhibit poor daily posture^10^, modifying it over the years. In other words, in seeking body balance, students' posture adapts to their lifestyle practices, and proper or improper posture habits lead to repercussions in adulthood^11^.

However, the use of any alternative postural evaluation instrument requires an evaluation of its psychometric properties^12^. Although the Flexicurve instrument is known for its use in the adult population^7^, it is necessary to verify the instrument's test-retest, intra-and inter-rater reliability prior to its use with children, because this population has distinct characteristics, such as thorax size and the sagittal curvatures of the spine. Therefore, the present study aimed to determine the test-retest, intra- and inter-rater reliability of the Flexicurve instrument for the evaluation of spinal curvatures in children.

## Method

### Sample

The sample size was calculated according to Walter et al.^13^ and Donner and Eliasziw^14^, assuming: the null hypothesis value of Intraclass Correlation Coefficient (ICC) to be 0.40 (e.g. on the basis that any value lower than .40 might be considered clinically "unacceptable"); 80% of power; two replicated measurements (one for each evaluator or twice by the same evaluator); and a significance level of 95% to detect an ICC value of .70 (based on previous literature^7^), a minimum of 33 participants was found. Allowing for losses, 40 children who had undergone X-ray examination in a hospital in Porto Alegre were invited to participate in the study. The inclusion criteria were children of both sexes, ranging in age from 5 to 15 years old. Children who had previous surgery or congenital deformity in spinal structures were excluded. This study was approved by the Research Ethics Committee of Universidade Federal do Rio Grande do Sul (UFRGS), Porto Alegre, RS, Brazil (number 19685), and the children's guardians signed the informed consent form.

### Evaluation protocol

The evaluation consisted of modeling the spine with the Flexicurve instrument, which provided the Flexicurve angles (FA) for the thoracic and lumbar spine. The same protocol was repeated in each evaluation: (1) on the child's bare back, the spinous processes (SP) of the C7, T1, T12, L1, L5, and S1 vertebrae were palpated and marked with stickers; (2) the child was in the standing position with normal posture; (3) elbows and shoulders were flexed to 90° and supported on the wall; (4) while remaining motionless; (5) the Flexicurve was molded to the child's back over the spine; (6) the Flexicurve was removed from the child's back and placed on graph paper, where the curvature was drawn and the SP marked; and (7) the FA was obtained using Biomec-FLEX free software (www.ufrgs.br/biomec), in which the input data consisted of the coordinate values representing the thoracic and lumbar curvatures, and the output data consisted of the curvature angles in the sagittal plane. The procedures (steps 1 to 7) were performed in accordance with the literature^7^.

### Design procedures

The spine postural evaluations using the Flexicurve instrument were performed by two previously trained independent evaluators (Ev1 and Ev2), with each subject being evaluated four times in two days. On the first day, there were two successive evaluations (Measure 1 and 2) by the same evaluator (Ev1) and a third evaluation (Measure 3) by a second evaluator (Ev2). After a seven-day interval, the children were re-evaluated (Measure 4) by one evaluator (Ev1). Both evaluators had at least two years' experience with postural evaluation of the spine and received 20 hours training in the use of the Flexicurve, which consisted of palpation, molding, transfer to paper, and analysis using the software.

For the test-retest reliability evaluation, data from the first evaluation (Measure 1) and from the second evaluation (Measure 2), performed successively by the same evaluator (Ev1), were used^15^. For the intra-rater reliability evaluation, data from the first evaluation (Measure 1) and from the evaluation performed by the same examiner (Ev1) seven days after (Measure 4) were used^15^. For the inter-rater reliability evaluation data from the first evaluation by Ev1 (Measure 1) and from the evaluation performed on the same day by Ev2 (Measure 3) were used^15^.

### Statistical analysis

The statistical analysis was performed with SPSS 17.0. Initially, a data descriptive analysis was carried out using descriptive statistics. The data normality was confirmed using the Shapiro-Wilk test. To verify the test-retest, intra- and inter-rater reliability, the Intra-Class Coefficient (ICC_2,2_), the Standard Error Measurement (SEM), and Minimum Detectable Change (MDC) were calculated. ICC_2,2_ was based on a 2-way (random effects) repeated-measures analysis of variance model with absolute agreement. The values found in the ICC were classified according to literature^16^ as weak (ICC<0.40), moderate (ICC between 0.40 and 0.75), and excellent (ICC>0.75). The Standard Error of the Measurement (SEM) was estimated using the following formula: SEM = SD, where SD is the standard deviation of the measurements. The Minimum Detectable Change (MDC) was estimated based on a 95% confidence interval, where MDC=1.96 * SEM. The level of significance adopted for all tests was 0.05.

## Results

Twenty-five (25) boys and 15 girls were evaluated (Table 1). The results for the test-retest reliability of thoracic kyphosis and lumbar lordosis angles expressed by ICC values were excellent, with SEM values less than 4.5° and MDC values less than 8.5° (Table 2).

**Table 1.** Anthropometric data of the sample (mean±SD).

**Sample**	**Age (years)**	**Body mass (kg)**	**Height (cm)**	**BMI (kg/cm^2^)**
Total (n=40)	10.2±2.8	39.3±12.9	1.4±0.2	19.5±2.8
Boys (n=25)	11.0±2.3	42.9±12.0	1.4±0.1	20.0±2.5
Girls (n=15)	9.1±3.3	33.5±12.5	1.3±0.2	18.7±3.2

BMI: Body mass index; SD: standard deviation.

**Table 2.** Results for test-retest, intra- and inter-rater reliability.

		**Mean±SD(°)**	**ICC_**2,2**_ (95% CI)**	***p*** **(ICC)**	**SEM(°)**	**MDC(°)**
Test-retest reliability						
Kyphosis (n=40)	Measure 1	37.5±9.3	0.93 (0.87-0.96)	<0.01	2.5	4.9
	Measure 2	37.5±9.5				
Lordosis (n=40)	Measure 1	26.0±9.5	0.80 (0.61-0.89)	<0.01	4.3	8.4
	Measure 2	26.2±9.5				
Intra-rater reliability						
Kyphosis (n=38)	Measure 1	36.0±9.9	0.82 (0.65-0.91)	<0.01	4.1	8.1
	Measure 4	36.4±9.5				
Lordosis (n=38)	Measure 1	24.8±9.5	0.67 (0.36-0.83)	<0.01	5.7	11.2
	Measure 4	26.0±10.4				
Inter-rater reliability						
Kyphosis (n=40)	Measure 1	35.7±9.0	0.83 (0.68-0.91)	<0.01	4.1	8.0
	Measure 3	37.6±10.8				
Lordosis (n=40)	Measure 1	25.2±9.5	0.72 (0.47-0.85)	<0.01	5.7	11.2
	Measure 3	27.2±12.0				

ICC: Intraclass Correlation Coefficient; SEM: Standard Error Measurement; MDC: Minimum Detectable Change; SD: standard deviation.

For the evaluation of intra-rater reliability, the results obtained by the ICC showed excellent and moderate levels for the angles of thoracic kyphosis and lumbar lordosis, respectively, with SEM values less than 6.0° and MDC values less than 11.5° (Table 2).

For the evaluation of inter-rater reliability, the results obtained by the ICC demonstrated excellent and moderate levels for the thoracic kyphosis and lumbar lordosis angles, respectively, with SEM values less than 6.0° and MDC values less than 11.5° (Table 2).

## Discussion

This study aimed to determine the test-retest, intra-and inter-rater reliability of the Flexicurve instrument for evaluating spinal curvatures in children. Acceptable correlation levels were found for the angles of thoracic kyphosis and lumbar lordosis. These results differ from those of Teixeira and Carvalho^6^, which showed only excellent levels of both inter- (ICC=0.94) and intra-rater reliability (ICC=0.87) for thoracic kyphosis in an adult population, and those of Oliveira et al.^7^, which also showed excellent levels of inter-rater reliability (ICC=0.94 for thoracic kyphosis; ICC=0.83 for lumbar lordosis) and intra-rater reliability (ICC=0.83 for thoracic kyphosis; ICC=0.78 for lumbar lordosis) in adults. Both studies used the Flexicurve instrument.

However, Letafatkar et al.^17^ evaluated the lumbar region with the Flexicurve instrument and found results that corroborate those of the present study, showing moderate intra-rater reliability (ICC ranging from 0.62 to 0.69) and inter-rater reliability (ICC=0.54). Lovell et al.^18^ also evaluated the lumbar region and found that the intra-rater reliability ranged from moderate to excellent (ICC 0.73 to 0.94) in addition to moderate inter-rater reliability, with ICC values of 0.41 and 0.50, which suggested that the evaluation of the lumbar region in adults with the Flexicurve may be viable if performed by the same person, but the degree of reproducibility may vary from evaluator to evaluator.

Dunleavy et al.^19^, who also investigated the inter-and intra-evaluator reliability of the Flexicurve using variable spine length and width, found that the measurements of the total length of the spine showed good intra-rater reliability (ICC=0.93), but the evaluation of the thoracic length, lumbar length, chest width, and lumbar width showed moderate intra-rater reliability (ICC=0.61-0.80). In addition, the inter-rater reliability for all measures was moderate (ICC=0.58 to 0.72), and the mean lengths indicated significant differences among the evaluators.

It is worth noting that, in all the evaluations of test-retest, intra- and inter-rater reliability of this study, the correlations were always lower in the lumbar region than in the thoracic region, demonstrating an inherent difficulty in evaluating this region. Previous studies have demonstrated the difficulty in evaluating the lumbar region. For example, Hinman^20^ evaluated the inter-rater reliability of the Flexicurve instrument by novice evaluators and found excellent correlation levels for thoracic kyphosis indices (ICC 0.93 and 0.94) and lower levels of correlation for lumbar lordosis (ICC of 0.60 and 0.73). The author noted that the difficulty in molding the Flexicurve to regions of smaller curvature or even concave features of the lumbar spine might have caused the greatest variability in lumbar measurements. Thus, Hinman's results^20^ are in accordance with the present study in that there is difficulty when evaluating smaller curvatures with the Flexicurve, particularly in the lower back. Another difficulty in evaluating the lumbar region is related to the palpation of anatomical landmarks in this region since the characteristics of the lumbar vertebrae hamper the identification and location of the spinal process^21^.

In the literature, several non-invasive instruments for exclusively evaluating thoracic kyphosis are described, a fact that is probably related to difficulties in the non-invasive evaluation of lumbar lordosis. For example, we can cite studies such as the one by Perriman et al.^22^, which verified the concurrent validity of the flexible electrogoniometer; the study by D'Osualdo et al.^23^, which validated the arcometer; and the study by Lewis and Valentine^24^, which determined the test-retest reliability of the inclinometer. All of these studies exclusively evaluated thoracic kyphosis.

In addition to the adequate levels of test-retest, intra- and inter-rater reliability obtained in this study, it is important to point out the variability inherent in the measurement in order to facilitate the correct interpretation of the results during clinical follow-up. Thus, the SEM values reflect the precision of the measurement, which in this study vary from 2.5° to 5.7°, depending on the region and the analysis conducted. These values can be considered clinically acceptable, since the variability found in the gold standard technique for measuring spinal curvature, the Cobb angle, is from 5° to 10°, for both intra- and inter-rater reliability^25^. Furthermore, it is also important to know what magnitude of the change in the measurement would be necessary to determine the existence of a real change rather than a mere measurement error^26^. Based on MDC values, it can be concluded that, when Flexicurve is used by the same evaluator or different evaluators during follow-up procedures in children, there needs to be a minimum of 8° to be considered a real change in the thoracic curvature and 11° in the lumbar curvature (Table 2).

MDC values around 10° suggest the instrument has poor sensitivity, which may be considered as a limitation for its use. On the other hand, it is important to point out that the SEM and MDC values are dependent on the variability among the subjects in the sample, which in this study was between 14° to 57° for thoracic curvature and 6° to 46° for lumbar curvature. Hence, in this study, the SEM and MDC values do not necessarily reflect the reliability of the measurements, but rather the variability of the sample. Nevertheless, restricted variability among the sample could also represent a limitation. Only two children in the sample presented increased thoracic curvature (over 50°)^27^^,^ 28, while none were found to have increased lumbar curvature (over 66.8°)^29^, which restricts the use of the Flexicurve in children in this range.

In summary, the test-retest, intra- and inter-rater reliability of the results presented in this study show that the Flexicurve instrument can be used in the initial evaluation of the spinal curvatures in children. However, for an instrument to be used for the purposes of diagnosis, it should be subjected to a validation process, and most studies on instrument validation are conducted with adult subjects, not children. Therefore, the Flexicurve still lacks concurrent validation in relation to gold standard technique for the detection of spinal alteration in order to ensure its diagnostic capacity.

## Conclusion

The Flexicurve evaluation method has test-retest, intra- and inter-rater reliability for the population of children between 5 and 15 years of age, which would allow its use in school screening to detect postural alterations at an early stage. However, to monitor spinal curvatures in the sagittal plane in children, complementary clinical measures are necessary.
